# X-ray Diffraction and Piezoelectric Studies during Tensile Stress on Epoxy/SbSI Nanocomposite

**DOI:** 10.3390/s22103886

**Published:** 2022-05-20

**Authors:** Marcin Godzierz, Bartłomiej Toroń, Piotr Szperlich, Piotr Olesik, Mateusz Kozioł

**Affiliations:** 1 Centre of Polymer and Carbon Materials, Polish Academy of Sciences, M. Curie-Skłodowskiej 34 Street, 41-819 Zabrze, Poland; 2 Centre for Science and Education, Institute of Physic, Silesian University of Technology, Krasińskiego 8 Street, 40-019 Katowice, Poland; bartlomiej.toron@polsl.pl (B.T.); piotr.szperlich@polsl.pl (P.S.); 3 Faculty of Materials Engineering, Silesian University of Technology, Krasińskiego 8 Street, 40-019 Katowice, Poland; piotr.olesik@polsl.pl (P.O.); mateusz.koziol@polsl.pl (M.K.)

**Keywords:** X-ray diffraction, SbSI nanowires, nanocomposite, piezoelectricity, strain sensor

## Abstract

In this paper, the performance of epoxy/SbSI nanocomposite under tensile stress was investigated. X-ray diffraction studies show the main stress mode has shear nature in the case of elastic deformation, while a combination of shear and tensile stress during plastic deformation caused lattice deformation of SbSI and shift of sulfur atoms along the *c* axis of the unit cell. Apart from that, the piezoelectric signals were recorded during tensile tests. Epoxy/SbSI nanocomposite responded to the applied tensile stress by generating a piezoelectric current with a relatively high value. The measured piezoelectric peak-to-peak current is relatively high (I_p-p_ = 1 pA) in comparison to the current flowing through the sample (8.16 pA) under an applied voltage of 100 V. The current level is independent of the deformation speed rate in contradistinction to complex stress states. The signal comes from the whole volume of the sample between electrodes and is generated by shear stress.

## 1. Introduction

### Highlights

- X-ray diffraction studies combined with tensile tests confirm that SbSI acts as reinforcement.
- A strong shift of sulfur atoms in SbSI unit cell under plastic deformation of epoxy/SbSI nanocomposite.
- Relatively high peak-to-peak current was measured (I_p-p_ = 1 pA) in elastic deformation.
- The piezoelectric response is independent of the deformation speed rate.

Deformation monitoring of composite structures is a very important issue, especially in the case of applications of polymer composites in aviation, aeronautics, and the automotive industry. The most frequently used structures and/or systems of strain gauges are mainly microchips, but also optical sensors and integrated piezoelectric sensors [1,2,3,4,5,6,7]. The last group of sensors has a particularly large application potential, due to the possibility of introducing piezoelectric material in the form of a powder into the resin and placing it in a specific layer of the laminate so that the generated current signal best describes the deformation of the structure [7,8,9]. The use of piezoelectric materials as structural elements of FRP (fiber reinforced polymer) composites allows for in situ monitoring of the current deformations of laminate structures [1,2,3,4,5,6,7].

One of the very promising candidates to be applied in composite strain sensors is antimony sulfoiodide (SbSI), which has been extensively examined over the past decades [9,10,11,12,13,14,15,16,17,18,19,20,21,22,23,24,25,26,27,28,29,30,31,32,33,34,35,36,37,38,39]. The antimony sulfoiodide (SbSI) is the main representative of the A^15^B^16^C^17^ class of ferroelectric semiconductors. It has a large number of interesting properties [40,41]. The investigation of SbSI started after the discovery of its photoconductivity [42] and its ferroelectric properties [10] in the early 1960s of the 20th century. The bulk antimony sulfoiodide (SbSI) in the ferroelectric phase crystallizes in the Pna2_1_ orthorhombic structure and is characterized by very high volume piezoelectric modulus d_v_ = 1 × 10^−9^ C/N [43] and electromechanical coupling coefficient k_33_ = 0.90 [13]. In a paraelectric phase i.e., above the Curie temperature, the SbSI structure rebuilds into Pnam. As a material with promising applications, SbSI single crystals were synthesized in a variety of ways. This material was obtained in a form of nanowires by using the sonochemical method. One can find the structure and the properties of SbSI nanowires in [23]. It shows not only excellent piezoelectric properties but also photoelectrical, pyroelectric, triboelectric, and ferroelectric, which allows it to be applied, eg. as gas sensors [33], humidity sensors [29], photoelectronic devices [34,35,39] or for water purification [30,31,38]. Previous studies focused on the fabrication and examination of polymer-based composites filled with SbSI nanowires showing that this type of functional composites can also be applied as, e.g., strain sensors in FRP structures [7,8,9,14,36], piezoelectric nanogenerators [26,36], energy harvesting or smart textiles [36,37].

An especially interesting application of SbSI nanowires as well as of other piezoelectric materials as BaTiO_3_ (BTO) [44,45,46,47,48,49], BiFeO_3_ (BFO) [50,51,52,53], K_0.5_Na_0.5_NbO_3_ (KNNO) [54,55,56], AlN [57], and PLZT-based (Pb_1-x_La_x_Zr_y_Ti_1-y_O_3_) ceramic [58,59,60,61] are the strain sensors in FRP, due to wide range of its applications in industry. However, proper design of piezoelectric integrated sensors in FRP requires precise interpretation of generated signal and its correlation with structure deformation. According to that, many different factors should be considered, but the most important one is the viscoelastic nature of the polymer matrix. The previous studies show that epoxy/SbSI sensors were characterized by different electric signals than pure SbSI [7,8,9,12,13,14], which is related to the presence of epoxy and its viscoelastic deformation. Another important factor is the orientation of nanowires, especially in the meaning of uniaxial tensile load, because highly oriented nanofibers will strongly increase the stiffness and tensile strength of composite [62,63,64,65,66,67,68,69,70]. However, even randomly oriented fibers increase the mechanical properties of various composites [62,63,64,65,66,67,68,69,70].

This paper presents the results of unique examination methods, which combine tensile loading, X-ray diffraction studies, and measurement of the piezoelectric response of epoxy/SbSI composite subjected to uniaxial tensile load. Combination of tensile load with XRD studies was performed for the first time and allows us to obtain very important data for the future design of integrated epoxy/SbSI composite strain sensor, in particular a correlation between applied stress mode, stress distribution in the sample, nanowires orientation, viscoelastic stress relaxation of the epoxy matrix, and measured signal.

## 2. Materials and Methods

### 2.1. Materials Fabrication

The first stage in the preparation of composite is the sonochemical fabrication of SbSI nanowires by the so-called Nowak’s method [23]. In this procedure, pure elements (antimony, sulfur, and iodine) were used to sonochemically obtain SbSI nanowires. The stoichiometric mixture of elements was placed in ethanol in a closed container made of polypropylene. It did not allow volatile synthesis products to escape. After that, the container was submerged in water in a cup-horn connected to ultrasonic processor VCX-750 with converter VC-334 (Sonics & Materials, Inc., Newtown, CT, USA). Refrigerated circulating bath AD07R (PolyScience, Niles, IL, USA) was used to keep a constant temperature of 293 K during the entire time (2 h) of the synthesis process. In the next step, the obtained gel was rinsed using pure ethanol and centrifuged to remove the remaining substrates. Finally, SbSI gel was inserted in a vacuum chamber under reduced pressure (5 mbar) at room temperature in order to evaporate ethanol from its volume. The SbSI nanowires in prepared xerogel have cross dimensions of tens of nanometers, with a medium value of 69 nm [39]. Their length reaches several microns. Further information on the sonochemical process, as well as on obtained SbSI xerogel, (e.g., SEM and HRTEM micrographs, SAED patterns, DRS spectrum) can be found in [23].

**Figure 1:**
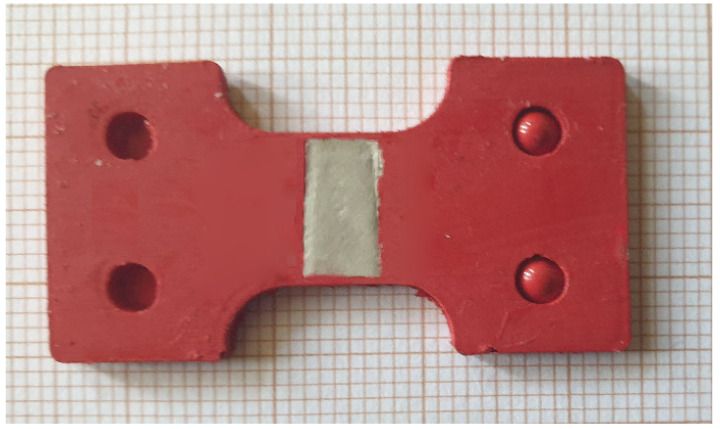
Photo of the sample with visible top electrode deposited for piezoelectric measurements.

The SbSI nanowires and LH288 epoxy resin (HAVEL COMPOSITES, Svesedlice, Czech Republic) were mixed in a 20% mass ratio to fabricate the composite. Then, they were pre-mixed mechanically and then mixed again in the ultrasonic bath. In the next step, the hardener H281 (HAVEL COMPOSITES, Svesedlice, Czech Republic) was added with a volume proportion to resin 1:4, according to technical requirements. The prepared mixture was placed in a silicone mold in form of 2 mm × 19 mm × 38 mm (thick × width × length), with 2 mm × 9.5 mm × 14.5 mm measurement base (Figure 1) and left in an Environmental Chamber SH-242 (Espec, Osaka, Japan) at a constant temperature (283 K) for 24 h to be cured. For piezoelectric measurements, the silver electrodes with dimensions of 9 mm × 5 mm were painted on both sides of the measurement base using high-purity silver paste 05002-AB (SPI Supplies) and copper wires were connected to them (Figure 1).

### 2.2. Evaluation Methods

XRD studies were performed using the D8 Advance diffractometer (Bruker AXS, Karlsruhe, Germany) with Cu-Kα cathode (λ = 1.54 Å) operating at 40 kV voltage and 40 mA current. The scan rate was 2°/min with scanning step 0.02° in the range of 10° to 120° 2Θ, resulting in a measurement time of about 55 min. Identification and fitting of the registered phase were performed using DIFFRAC.EVA program with use of ICDD PDF#4 database, while exact peak shapes, lattice parameters, crystallite size, lattice strain, and scale were refined simultaneously using Rietveld refinement [71,72,73] in TOPAS 6 program, based on Williamson–Hall theory. After convergence, atomic positions and finally isotropic temperature factors were included in the refinement. The pseudo-Voigt function was used in the description of diffraction line profiles at the Rietveld refinement [71,72]. The R_wp_ (weighted-pattern factor) and GOF (goodness-of-fit) parameters were used as numerical criteria for the fitting quality of experimental diffraction data.

Stress-XRD analysis was performed using DEBEN microtensile stage mounted at D8 Advance diffractometer with use of iso-inclination mode (Bruker, Karlsruhe, Germany) based on (411) and (530) peaks of SbSI phase with the nominal position at 48.98° and 60.84° of 2θ pattern, respectively, according to EN-15305 standard [74]. In tensile tests, a 2 kN crosshead was used, moving with a speed of 0.4 mm/min. For residual stress analysis, the following materials parameters were used: Young’s modulus 31 GPa and Poisson ratio 0.26, which gives S_1_ = −8.387 × 10^−6^ MPa and 1/S_2_ = 4.065 × 10^−5^ MPa^−1^.The 2.5 MPa limit was used for a stress-free material, while the 1.25 MPa limit was used for a shear stress contribution. After preliminary tensile tests, the stress analysis in SbSI nanowires was performed using different applied loads, corresponding to various displacements of samples (Figure S1). For stress analysis, the standard fit was used in (411) peak position fitting, while for (530) peak gravity fit was used. Applied stress mode was established as normal with shear stress contribution. Residual stress analysis was used in two configurations—up to composite break and for elastic deformation only. Scanning electron microscopy studies of obtained fractures were performed using Quanta FEG 250 (ThermoFischer Scientific, Waltham, MA, USA) using 5 kV voltage and low vacuum mode.

Piezoelectric response of epoxy/SbSI composites was determined by uniaxial tensile test using Instron 4469 testing machine with two electrodes in a sandwich configuration. The electric signal was registered by Keithley 6517A electrometer (Keithley Instruments, Cleveland, OH, USA). The electric response of the sample was continuously measured during the test. The sample was stretched by 0.025 mm successively for the loading bar speeds assumed at 1, 2, 5, 10, 20, 50, 100, 200, and 500 mm/min. The maximum detected force was in the range of 115–130 N and decrease to approximately 80 N after 60 s for every deformation speed. The waiting period of 60 s until a constant value of the measured voltage was established (sample loading) during the voltage measurement and then the system returned to its initial state (sample unloading). The 60 s time was based on preliminary tests for small deflections and low strain rates. It guaranteed that the value of the recorded signal was fixed.

## 3. Results and Discussion

### 3.1. Uniaxial Tensile Tests and X-ray Diffraction Studies

**Figure 2:**
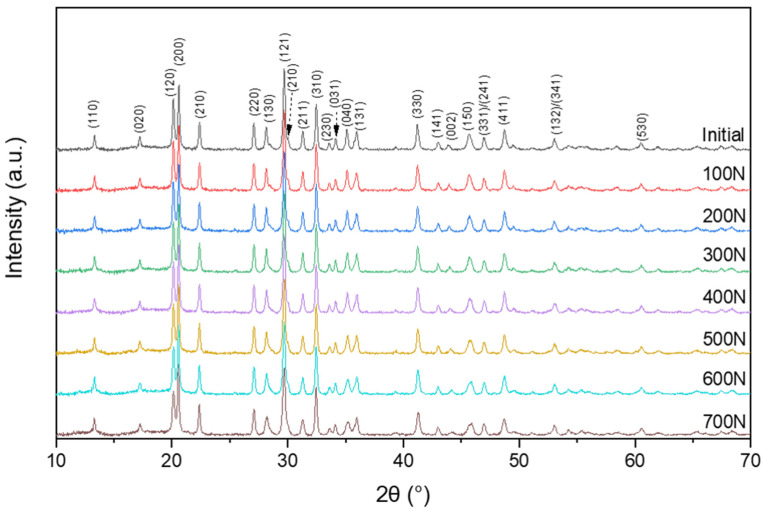
X-ray diffraction patterns of epoxy/SbSI samples at different loads with marked main peaks of orthorhombic SbSI phase.

**Figure 3:**
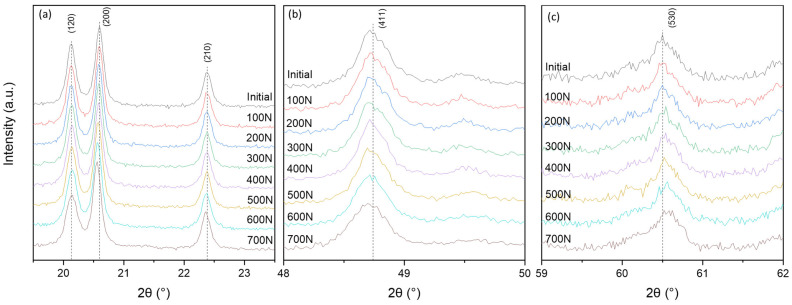
Peak shift of interplanar spacing observed under different loads: (**a**) (120), (200) and (210) peaks, (**b**) (411) peak and (**c**) (530) peak; dashed lines indicate peak position of the unloaded sample.

All peaks in the registered X-ray diffraction patterns (Figure 2) are identified as SbSI with an orthorhombic Pnam structure (PDF#01-075-0781). However, some minor changes in peak positions under different loads were detected, as can be seen in Figure 3 and Table S1. The typical peaks shift under tensile load should be toward lower angles direction, corresponding to higher interplanar spacing and confirming that SbSI nanowires act as reinforcing fibers in an epoxy matrix and are stretched (Figure 3a,b, Table S1). However, the viscoelastic nature of the epoxy matrix is also visible on X-ray diffraction patterns in form of peaks shifted toward higher angles direction, corresponding to lower interplanar spacing. It shows that epoxy matrix sheared SbSI nanowires (Figure 3c, Table S1) [8,64,65,75,76,77]. One can see that the 2θ angle of (530) peak position is about 60.5°. It results in about a 25 min delay time between the beginning of stress application and (530) peak measurement considering the applied scan rate (2°/min), which might explain the peak shift in higher angles direction due to strain relaxation.

**Figure 4:**
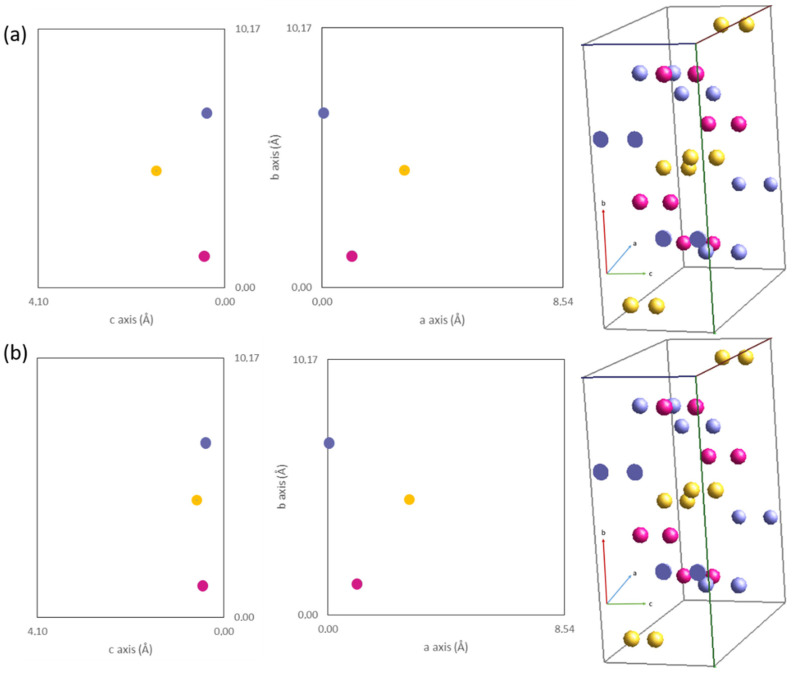
Schematic representation of SbSI atomic position in Pnam orthorhombic lattice: in unloaded composite (**a**) and under 400 N tensile load (**b**); ●—antimony atoms, ●—sulfur atoms, and ●—iodine atoms.

Rietveld refinement (Table S2, Section S1) shows that the application of various loads slightly changes the lattice parameters and volume, especially b-axis length, which becomes smaller by about 0.03 Å under 700 N load in comparison to the nonstressed sample. Moreover, lattice strain increased from 0.27% up to 0.48%. Crystallite size does not significantly change with the mean value of approximately 80 nm. Atomic positions of antimony, sulfur, and iodine calculated using Rietveld refinement are almost stable up to 300 N load (see Supplementary Materials, Section S1), but for over 400 N loads the strong shift of the sulfur atom was detected, which is presented in Figure 4. This shift is in the c-axis direction, which might suggest a strong interaction between atoms in the unit cell, especially considering the atomic radii of sulfur [14,15,16,20,78,79]. It might be a result of viscoelastic interactions between the epoxy matrix and SbSI nanowires as well as an effect of shear stress presence, but its nature is not clear and requires additional studies on SbSI single crystals or polycrystalline SbSI samples under various loads, considering tensile, compressive, and more complex stress states. However, to obtain more clear results, residual stress analysis studies were performed, as was established in the evaluation methods paragraph.

In Figure 4 one can see that atoms shift in a lattice under applied stress. It is especially visible on sulfur atoms, in which the shift is significant due to its small molar mass compared with iodine and antimony and the interaction between them [80,81]. The sulfur atoms shift along the c-axis direction. This explains the anisotropy of piezoelectric response in SbSI crystals, which is characterized by the best piezoelectric and other physical properties along the c-axis [12].

### 3.2. Stress Analysis up to Break Using (411) Peak

Typical residual stress diagrams are shown in Figure S2, while values of linear and shear stress calculated for SbSI nanowires in epoxy resin are listed in Table 1. It can be seen that with the increase in applied tensile load, the linear stress increases from stress-free material up to 120 MPa. This is possible only due to the nanometric cross-section of SbSI nanowires. Shear stress shows a similar tendency, with an increase from 11 to approximately 30 MPa at 600 N load. High values of shear stress are the result of SbSI nanowires’ random orientation in the epoxy matrix, but in the initial state, it is most likely an effect of epoxy shrinkage during the curing process. Detected stress values for SbSI nanowires are much higher than the applied load to composite; however, nanowires act as short fibers in this case, and the load applied by them is much higher than for composite due to their low cross-sectional dimensions.

However, the elastic deformation region of epoxy/SbSI composite is in the load range from 0 to about 150 N; thus, calculated stress refers not only to the stretching of SbSI nanowires but also to shear interactions between epoxy matrix and nanowires [8,64,65,75,76,77]. According to that, further tests were performed, in the elastic deformation region of examined composite.

### 3.3. Stress Analysis in the Elastic Region Using (411) and (530) Peak

Results of stress evaluation in the elastic region show that the dependence of tensile stress distribution on applied stress is linear in the range from 60 N load up to 150 N load, independent of the chosen peak (Table 2). However, the (411) peak shows lower values of shear stress contribution than the (530) peak, especially under higher loads. High values of linear stress detected for 120 N and 150 N loads suggest a partial contribution of epoxy deformation and its interaction with SbSI nanowires [8,75,76,77].

### 3.4. Piezoelectric Response of the Composite Sensor

**Figure 5:**
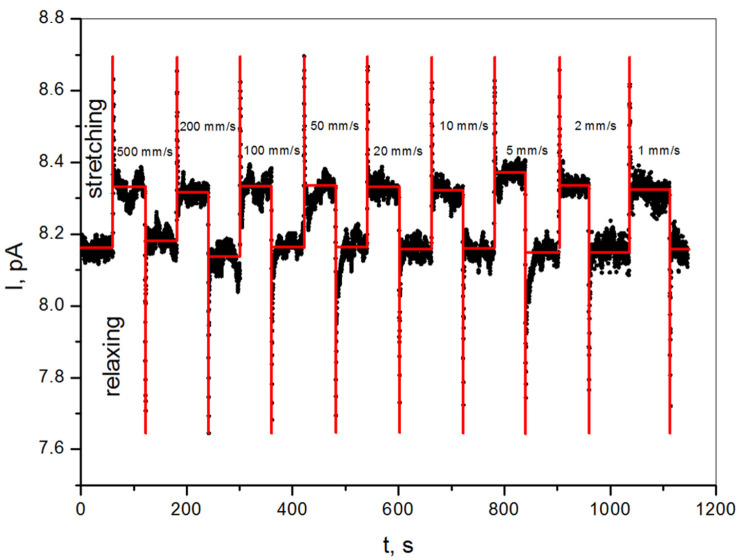
Current flowing through the sample under 100 V of applied voltage, registered during the non-destructive static tensile tests at a constant deformation of 0.025 mm, with a stretching rate of 1–500 mm/min (black points—measurement data; red line—expected ideal characteristic).

The piezoelectric current response for non-destructive static tensile tests at a constant deformation of 0.025 mm and an increasingly higher crosshead speed rate in the range of 1–500 mm/min is presented in Figure 5.

One can notice that the current flowing through the sample under an applied voltage of 100 V is 8.16 pA. It allows calculating the resistivity of epoxy/SbSI composite considering the dimension of the sample. The calculated volume resistivity is ρ = 2.74 × 10^11^ Ωm.

During tensile tests, the measurable signals were recorded, which were independent of various deformation speed rates (Figure 5). Most likely, it is an effect of applied voltage resulting in current flow. In this case, the piezoelectric response of the sample results in charge generation, i.e., piezoelectric current, but it is not dependent on the applied speed rate.

**Figure 6:**
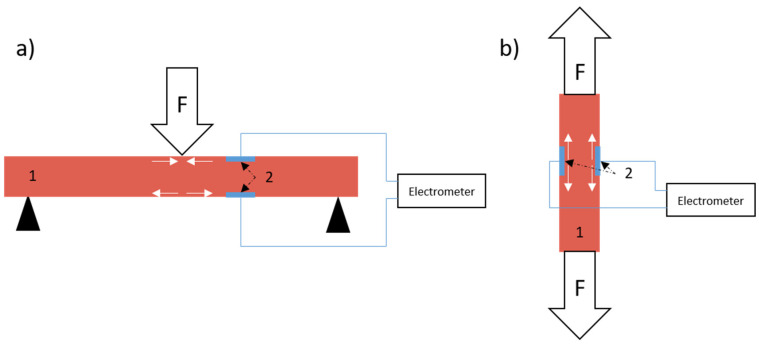
Schematic representation of stress distribution near electrodes in different loading conditions: (**a**) bending test and (**b**) tensile test; 1—epoxy/SbSI composite, 2—electrodes, F—direction of applied force, white arrows indicate stress distribution near electrodes during mechanical tests in elastic deformation region.

**Figure 7:**
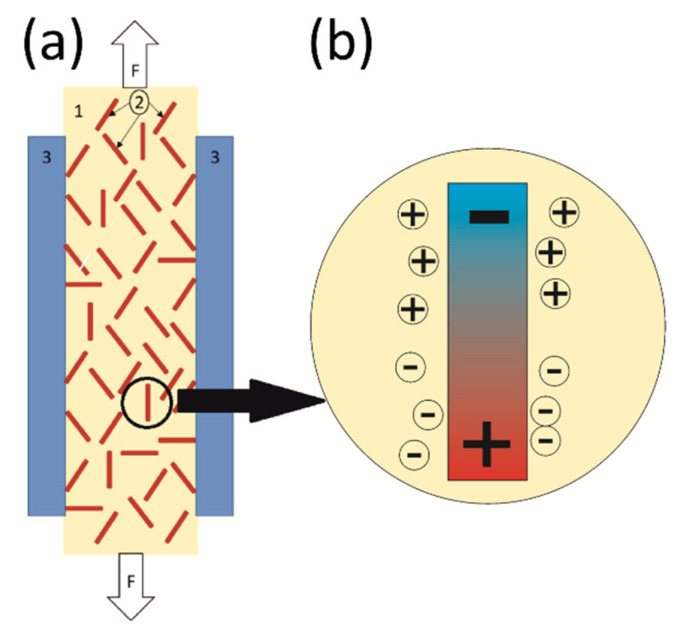
Schematic representation of epoxy/SbSI nanocomposite (**a**), and scheme of piezoelectric response (**b**) under tensile stress; 1—epoxy matrix, 2—SbSI nanowires, 3—electrodes; white arrows in (**a**) indicate the force direction.

Due to the high resistivity of the epoxy/SbSI composite, the measured piezoelectric peak-to-peak current is relatively high (I_p-p_ = 1 pA) in comparison to the current flowing through the sample (8.16 pA) under an applied voltage of 100 V. The signal comes from the whole volume of the sample between electrodes and is generated by shear stress in SbSI nanowires during tensile tests [8,75,76,77]. Moreover, during tensile tests in a sandwich configuration, both electrodes are in a similar stress mode, unlike in bending tests (Figure 6). One can see the rapid growth of piezoelectric current under shear stress (Figure S2). At this time, every nanowire act as a dipole due to lattice deformation and ions displacement (Figure 4). Electrons and holes flow through the sample volume in order to shield these dipoles (Figure 7b). The charge transfer is visible as maxima in the current characteristic (Figure 5). The charges run in the opposite direction during relaxation, which is apparent as minima in Figure 5. In our previous research [7,8,9] only bending tests were applied, where one of the electrodes was on the top of the sample and the second one was on the bottom, as shown in Figure 6a. It additionally allows the generation of a difference in electronic charge due to differences in stress distribution and generates a measurable voltage or current as a result. A comparison of both tests allows us to state that this type of composite piezoelectric sensor with randomly oriented SbSI nanowires generates a higher signal during a complex stress state, such as bending.

Similar observations were reported by Purusothaman et al. [82], where authors measured the negligible response of the PMMA/SbSI composite and SbSI micro-rods sensors under tensile stress, while weaker responses were detected for compressive stress and stronger for bending. However, the authors used polycrystalline SbSI rods fabricated by solid-state reaction with micrometric size, but the general pattern is convergent. Similar studies have been performed in past years for sensors based on ZnO nanorods [83,84,85], laminated PZT thin films [86], Bi_4_Ti_3_O_12_ nanoparticles [87], Si/SiO_2_ nanowires [88], and BN nanotubes [89] which show that bending or compressive stress is more favorable than the tensile one.

As described above, features of epoxy/SbSI nanocomposite allow for its application not only as a strain sensor but also in smart sensing [26,87,88,89,90] and energy harvesting [9,36,37,76] applications, due to the extraordinary piezoelectric constant of SbSI [13,14]. Moreover, the usage of the epoxy matrix allows for obtaining almost all possible shapes of the nanocomposite, which due to its easy formability, is typical for them. As the result of the fabrication procedure, prepared samples belong to the so-called 0–3 group of nanocomposites [91] since SbSI nanowires are randomly dispersed in an epoxy matrix (see scheme in Figure 7a).

### 3.5. Scanning Electron Microscopy Studies

**Figure 8:**
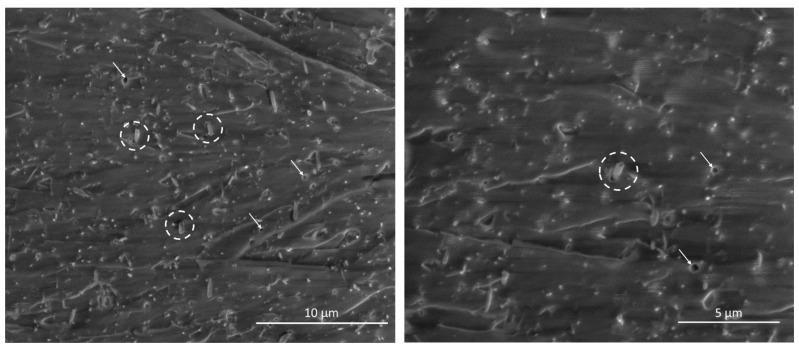
SEM micrographs of epoxy/SbSI nanowires fracture after tensile test; dashed lines indicate visible cracks at epoxy/SbSI interface, while white arrows pore with morphology similar to SbSI nanowires cross-section.

Fractures of epoxy/SbSI nanocomposite sample broken during measurements at 686 N are presented in Figure 8. The presented broken interfaces are normal for the applied force direction. It can be seen that nanowires are well dispersed in the epoxy matrix and randomly oriented, as was described in previous works [7,8,9,36]. Part of the SbSI nanowires was ripped out from epoxy or broken during tensile stress. Some minor pores are also visible, with shapes and sizes similar to SbSI nanowires. Some of the nanowires were also untouched, with visible cracks at the epoxy/reinforcement interface. It suggests the partial orientation of SbSI nanowires during tensile stress, due to the viscoelastic nature of the epoxy matrix, resulting in pores formation at the interface, and explains the untouched structure and presence of ripped out nanowires and pores with similar sizes and shapes.

## 4. Conclusions

Results from epoxy/SbSI nanowires integrated piezoelectric strain sensor has been presented. As a novel approach, structural changes in SbSI morphology as composite reinforcement under various loads were presented and residual stress in SbSI nanowires was calculated. Performed studies allow us to formulate the following conclusions:

1. Rietveld refinement of XRD patterns obtained at various loads (in the range of 0–700 N) shows that *a* and *b* lattice parameters of SbSI unit cell slightly decrease, while the *c* parameter remains constant. It results in a slight decrease in lattice volume, but also results in a relatively high lattice strain, especially under plastic deformation of composite. Moreover, the strong shift of sulfur atoms was detected under loads higher than 400 N, which is responsible for the anisotropy of the piezoelectric properties of SbSI.
2. Major stress that affects SbSI nanowires in the epoxy matrix has shear nature, due to its random orientation in a matrix. Moreover, two other types of stress were detected in SbSI nanowires—the tensile one, which is present during tensile tests, and slight compression, regarding the viscoelastic nature of the epoxy matrix.
3. This type of composite piezoelectric strain sensor with randomly oriented SbSI nanowires requires a complex stress state, such as bending. Uniaxial stress, especially in the elastic deformation range, requires external voltage to measure the piezoelectric response due to the high impedance and resistivity (ρ = 2.74 × 10^11^ Ωm) of the sample.
4. Epoxy/SbSI nanocomposites driven with an applied voltage respond to the applied tensile stress by generating a piezoelectric current with a relatively high value (8.16 pA, I_p-p_ = 1 pA). The current level is independent of the deformation speed rate in contradistinction to complex stress states. Piezoelectric measurements may incline this material as potentially applicable in a piezoelectric strain sensor as well as smart sensing materials.

## Figures and Tables

**Table 1:** Stress values calculated for SbSI nanowires using residual stress analysis under different loads; sample broke at 686 N.

Step	Linear Stress, MPa	Shear Stress, MPa
Initial	−0.4 (2) *	11 (2)
100 N	18 (3)	12 (2)
200 N	39 (21)	14 (2)
300 N	66 (17)	19 (1)
400 N	100 (20)	22 (3)
500 N	110 (33)	27 (2)
600 N	120 (37)	29 (3)

* No linear stress in the material.

**Table 2:** Values of residual stress analysis in elastic deformation region using various peaks.

Step	(411) Peak		(530) Peak	
	Linear Stress, MPa	Shear Stress, MPa	Linear Stress, MPa	Shear Stress, MPa
Initial	−0.4 (2) *	11 (2)	0.3 (4) *	8 (4)
30 N	6 (4)	12 (3)	4 (3)	13 (3)
60 N	12 (3)	15 (2)	21 (4)	13 (3)
90 N	12 (3)	16 (2)	22 (3)	16 (5)
120 N	20 (6)	18 (3)	24 (5)	19 (5)
150 N	27 (5)	19 (3)	27 (2)	26 (4)

* No linear stress in the material.

## Data Availability

Not applicable.

## Supplementary Materials

The following supporting information can be downloaded at: https://www.mdpi.com/article/10.3390/s22103886/s1. Figure S1. Force vs. displacement curves of epoxy/SbSI composite obtained during a static tensile test (red line) and in situ XRD tensile test (black dashed line); Table S1. Peak position determined for peaks visible at Figure 3; Table S2. Lattice evolution of SbSI orthorhombic phase in epoxy/SbSI composite during tensile test; Figure S2. Typical residual stress diagram obtained for SbSI nanowires in epoxy-based composites under different applied loads; Section S1. Results of Rietveld refinement of epoxy/SbSI nanocomposite under various loads.
