# Supplementary material for: X-ray Diffraction and Piezoelectric Studies during Tensile Stress on Epoxy/SbSI Nanocomposite
Source: Sensors (Basel). 2022 May 20;22(10):3886. doi: 10.3390/s22103886 (PMC9145287; doi:10.3390/s22103886)
Supplement: Supplementary file 1 [file sensors-22-03886-s001.zip › sensors-1734251-supplementary.pdf]

Supplementary materials to:

X-ray diffraction and piezoelectric studies during tensile stress on epoxy/SbSI nanocomposite

Marcin Godzierz[1,*], Bartłomiej Toroń[2,*], Piotr Szperlich[2], Piotr Olesik[3], Mateusz Kozioł[3,*]

1 - Polish Academy of Sciences, Centre of Polymer and Carbon Materials, M. Curie-Skłodowskiej 34 street, 41-819 Zabrze, Poland

2 - Silesian University of Technology, Institute of Physic - Centre for Science and Education, Krasińskiego 8 street, 40-019 Katowice, Poland

3 - Silesian University of Technology, Faculty of Materials Engineering, Krasińskiego 8 street, 40-019 Katowice, Poland

Corresponding authors: mgodzierz@cmpw-pan.edu.pl, bartlomiej.toron@polsl.pl, mateusz.koziol@polsl.pl

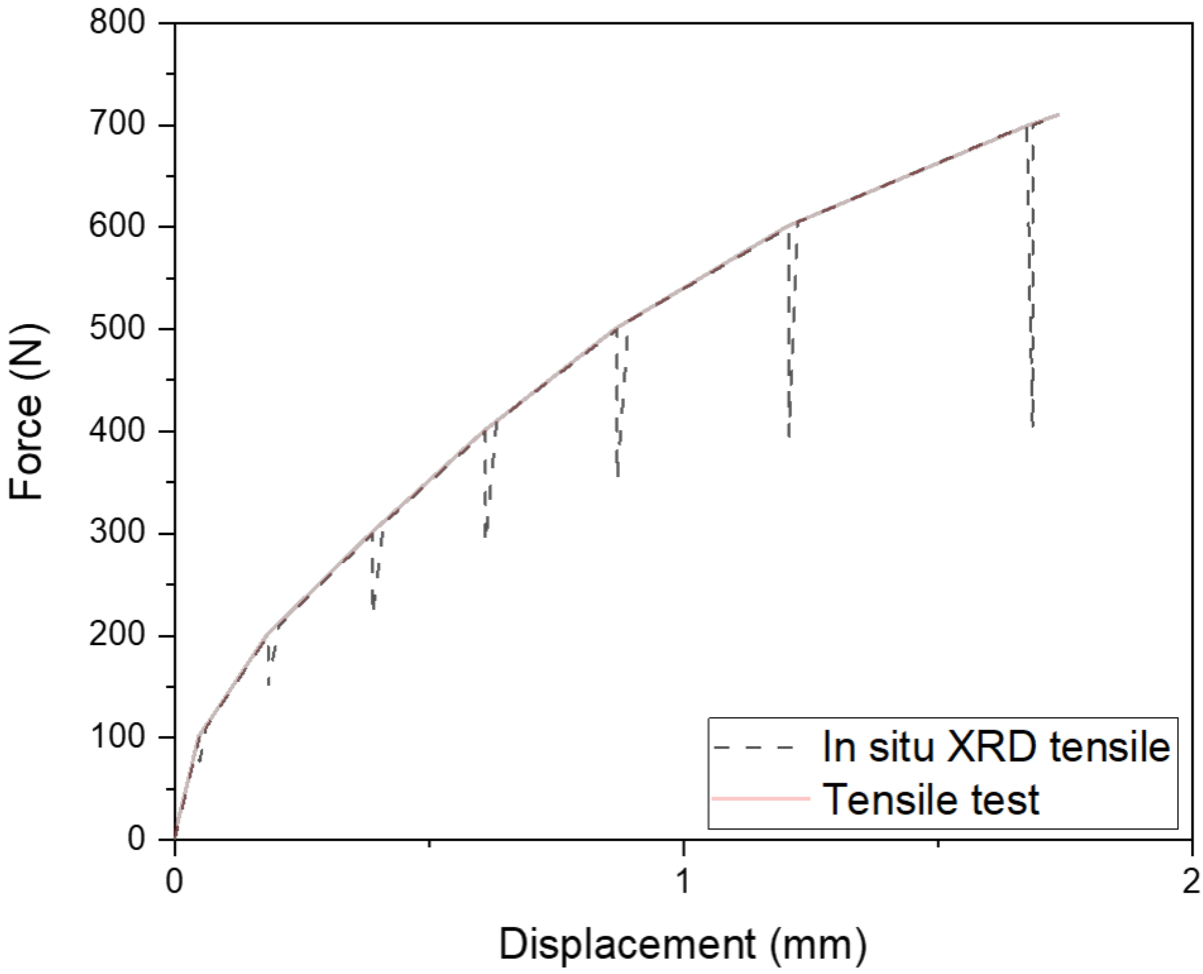

Figure S1. Force vs. displacement curves of epoxy/SbSI composite obtained during a static tensile test (red line) and *in situ* XRD tensile test (black dashed line)

Table S1. Peak position determined for peaks visible at Figure 3.

	Peak position 2θ, °				
	(120) peak	(200) peak	(210) peak	(411) peak	(530) peak
Nominal (ICDD)	20.36803	20.82288	22.61120	48.96762	60.80179
Initial	20.35697	20.78625	22.57694	48.88122	60.64761
100N	20.34963	20.78843	22.57716	48.88797	60.65369
200N	20.34008	20.78860	22.57515	48.88880	60.66514
300N	20.33168	20.78544	22.57051	48.88208	60.67654
400N	20.32503	20.78554	22.56910	48.88131	60.68227
500N	20.31979	20.78264	22.56540	48.87474	60.69712
600N	20.31371	20.78005	22.56177	48.86731	60.70528
700N	20.31061	20.77862	22.55984	48.86491	60.70684

Table S2. Lattice evolution of SbSI orthorhombic phase in epoxy/SbSI composite during tensile test

Step	Lattice parameters, Å	Lattice volume, $Å^3$	Lattice strain, %	Crystallite size, nm
Initial	a = 8.543, b = 10.168, c = 4.102	356.34	0.27(6)	83(5)
100N	a = 8.542, b = 10.166, c = 4.102	356.27	0.28(6)	85(5)
200N	a = 8.541, b = 10.162, c = 4.102	356.04	0.28(6)	83(3)
300N	a = 8.540, b = 10.159, c = 4.102	355.86	0.29(8)	81(4)
400N	a = 8.540, b = 10.155, c = 4.102	355.70	0.32(7)	83(5)
500N	a = 8.539, b = 10.149, c = 4.102	355.47	0.36(5)	83(5)
600N	a = 8.539, b = 10.143, c = 4.102	355.31	0.41(7)	80(7)
700N	a = 8.539, b = 10.138, c = 4.102	355.27	0.48(7)	79(8)

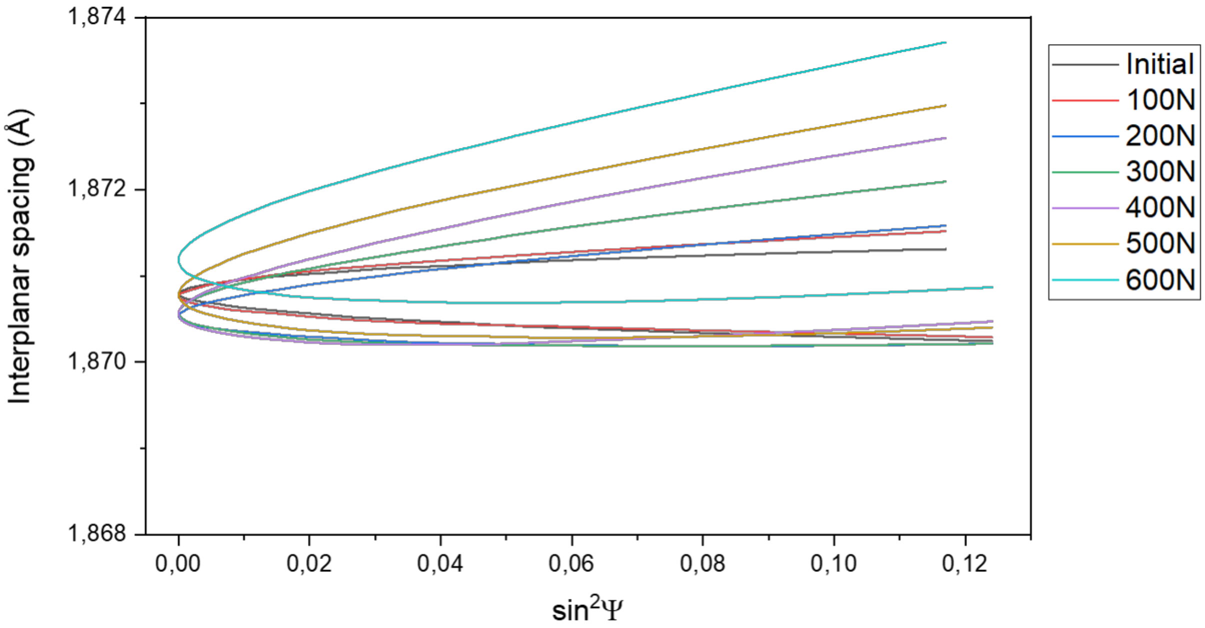

Figure S2. Typical residual stress diagram obtained for SbSI nanowires in epoxy-based composites under different applied loads

Section S1. Results of Rietveld refinement of epoxy/SbSI nanocomposite under various loads:

Initial:

Phase name	SbSI
R-Bragg	1.925
Spacegroup	Pnam
Scale	0.0001(2)
Cell Mass	2000(2000)
Cell Volume (Å^3)	356.337(3)
Wt% - Rietveld	100.000
Double-Voigt|Approach	
Cry size Gaussian	98(3)
k: 1 LVol-IB (nm)	92(3)
k: 0.89 LVol-FWHM (nm)	82.5(2)
Strain	
Strain L	0.266(10)
e0	0.00079(2)
Crystal Linear Absorption Coeff. (1/cm)	3000(3000)
Crystal Density (g/cm^3)	10(11)
Lattice parameters	
a (Å)	8.5430(4)
b (Å)	4.1021(2)
c (Å)	10.168(5)

Site	Np	x	y	z	Atom	Occ	Beq
SB1	8	0.1231(3)	0.1072(9)	0.1211(3)	Sb	1.0(16)	0.5
S1	8	0.3426(11)	0.366(4)	0.4514(8)	S	1.0(16)	0.5
I1	8	0.0073(3)	0.0948(8)	0.6731(2)	I	1.0(16)	0.5

Rexp : 2.44 Rwp : 3.83 Rp : 2.76 GOF : 1.57

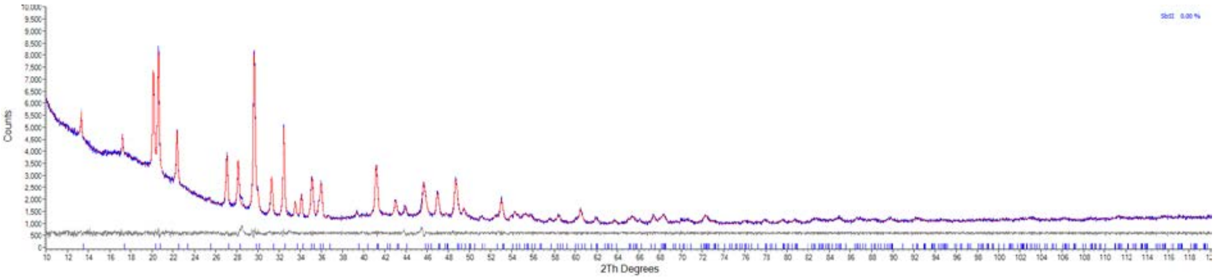

Figure S3. Fitted X-ray diffraction pattern of unloaded epoxy/SbSI nanocomposite; blue line represent experimental data, red one – simulated curve, grey line – differential curve; blue ticks represents peaks position of orthorhombic SbSI Pnam structure

100N:

Phase name	SbSI
R-Bragg	1.871
Spacegroup	Pnam
Scale	0.0000726(2)
Cell Mass	2251(5)
Cell Volume (Å^3)	356.268(3)
Wt% - Rietveld	100.000
Double-Voigt|Approach	
Cry size Gaussian	98(3)
k: 1 LVol-IB (nm)	92(3)
k: 0.89 LVol-FWHM (nm)	84.7(2)
Strain	
Strain L	0.278(10)
e0	0.00079(2)
Crystal Linear Absorption Coeff. (1/cm)	2660(5)
Crystal Density (g/cm^3)	10.51(2)
Lattice parameters	
a (Å)	8.5420(4)
b (Å)	4.1020(2)
c (Å)	10.166(5)

Site	Np	x	y	z	Atom	Occ	Beq
SB1	8	0.1230(3)	0.1068(9)	0.1210(3)	Sb	0.986(3)	0.5
S1	8	0.3429(10)	0.369(4)	0.4521(8)	S	0.982(12)	0.5
I1	8	0.0075(3)	0.0949(8)	0.6731(2)	I	1.023(3)	0.5

Rexp : 2.46 Rwp : 3.85 Rp : 2.77 GOF : 1.57

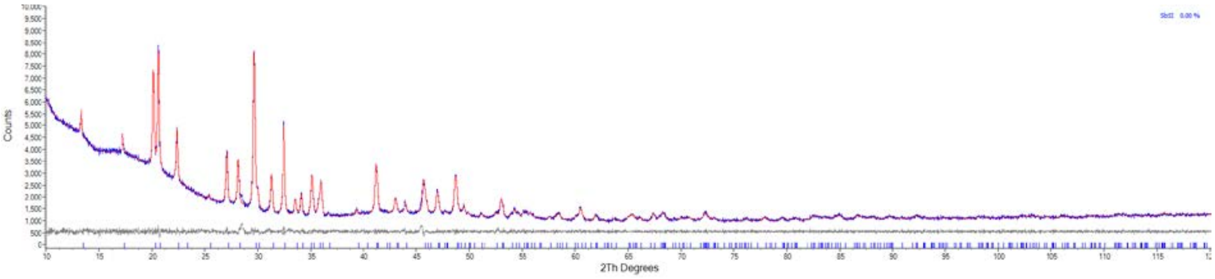

Figure S4. Fitted X-ray diffraction pattern of epoxy/SbSI nanocomposite under 100N load; blue line represent experimental data, red one – simulated curve, grey line – differential curve; blue ticks represents peaks position of orthorhombic SbSI Pnam structure

200N:

Phase name	SbSI
R-Bragg	2.314
Spacegroup	Pnam
Scale	0.0000691(2)
Cell Mass	2234(5)
Cell Volume (Å^3)	356.035(3)
Wt% - Rietveld	100.000
Double-Voigt|Approach	
Cry size Gaussian	95(4)
k: 1 LVol-IB (nm)	93(3)
k: 0.89 LVol-FWHM (nm)	83(4)
Strain	
Strain G	0.281(9)
e0	0.000928(19)
Crystal Linear Absorption Coeff. (1/cm)	2643(5)
Crystal Density (g/cm^3)	10.42(2)
Lattice parameters	
a (Å)	8.5409(4)
b (Å)	4.1019(2)
c (Å)	10.1620(5)

Site	Np	x	y	z	Atom	Occ	Beq
SB1	8	0.1232(3)	0.1125(9)	0.1215(3)	Sb	0.979(3)	0.5
S1	8	0.3458(11)	0.372(4)	0.4547(8)	S	0.953(13)	0.5
I1	8	0.0078(3)	0.0950(8)	0.6733(2)	I	1.020(3)	0.5

Rexp : 2.44 Rwp : 3.87 Rp : 2.82 GOF : 1.59

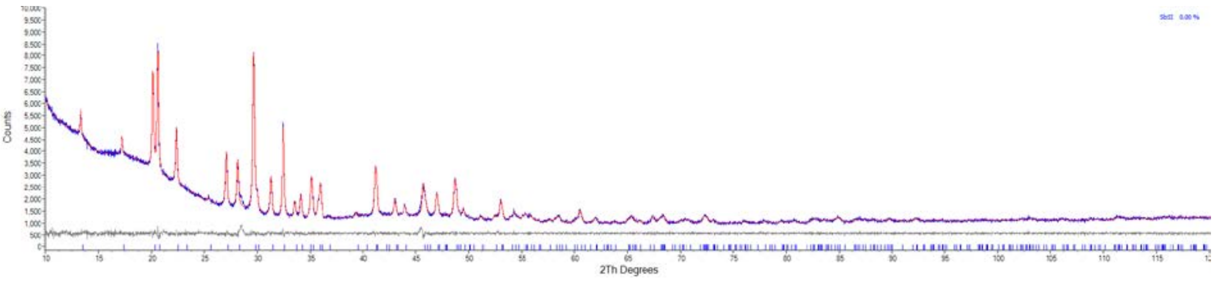

Figure S5. Fitted X-ray diffraction pattern of epoxy/SbSI nanocomposite under 200N load; blue line represent experimental data, red one – simulated curve, grey line – differential curve; blue ticks represents peaks position of orthorhombic SbSI Pnam structure

300N:

Phase name	SbSI
R-Bragg	2.483
Spacegroup	Pnam
Scale	0.0000696(2)
Cell Mass	2233(5)
Cell Volume (Å^3)	355.861(3)
Wt% - Rietveld	100.000
Double-Voigt|Approach	
Cry size Gaussian	92(5)
k: 1 LVol-IB (nm)	87(3)
k: 0.89 LVol-FWHM (nm)	81.2(4)
Strain	
Strain G	0.294(9)
e0	0.001101(19)
Crystal Linear Absorption Coeff. (1/cm)	2646(6)
Crystal Density (g/cm^3)	10.44(3)
Lattice parameters	
a (Å)	8.5401(5)
b (Å)	4.1019(2)
c (Å)	10.1587(6)

Site	Np	x	y	z	Atom	Occ	Beq
SB1	8	0.1231(3)	0.1132(9)	0.1219(3)	Sb	0.974(3)	0.5
S1	8	0.3478(11)	0.363(4)	0.4582(8)	S	0.962(13)	0.5
I1	8	0.0072(3)	0.0939(8)	0.6732(2)	I	1.022(3)	0.5

Rexp : 2.49 Rwp : 4.13 Rp : 2.92 GOF : 1.66

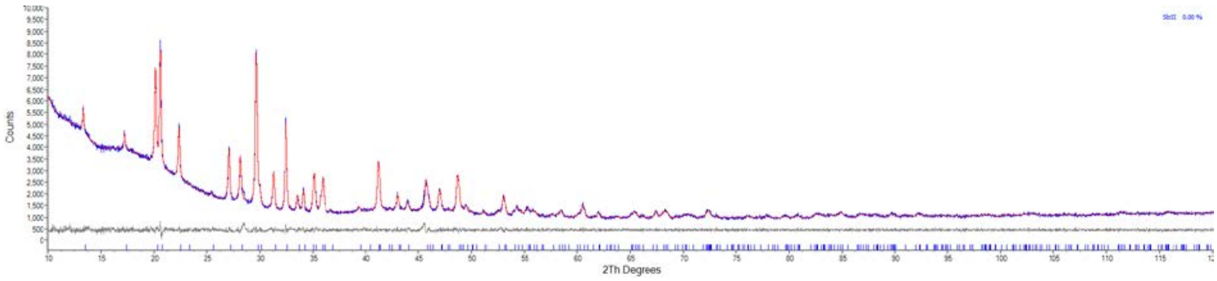

Figure S6. Fitted X-ray diffraction pattern of epoxy/SbSI nanocomposite under 300N load; blue line represent experimental data, red one – simulated curve, grey line – differential curve; blue ticks represents peaks position of orthorhombic SbSI Pnam structure

400N:

Phase name	SbSI
R-Bragg	2.160
Spacegroup	Pnam
Scale	0.000076(9)
Cell Mass	2260(90)
Cell Volume (Å^3)	355.699(4)
Wt% - Rietveld	100.000
Double-Voigt|Approach	
Cry size Gaussian	88(3)
k: 1 LVol-IB (nm)	85(3)
k: 0.89 LVol-FWHM (nm)	83.4(3)
Strain	
Strain L	0.318(14)
e0	0.00111(3)
Crystal Linear Absorption Coeff. (1/cm)	2670(110)
Crystal Density (g/cm^3)	10.6(4)
Lattice parameters	
a (Å)	8.5383(6)
b (Å)	4.1020(3)
c (Å)	10.1552(7)

Site	Np	x	y	z	Atom	Occ	Beq
SB1	8	0.1228(4)	0.1104(11)	0.1219(4)	Sb	0.97(6)	0.5
S1	8	0.3447(12)	0.144(5)	0.4614(9)	S	1.02(6)	0.5
I1	8	0.0057(4)	0.0963(9)	0.6731(3)	I	1.03(6)	0.5

Rexp : 2.55 Rwp : 4.61 Rp : 3.25 GOF : 1.81

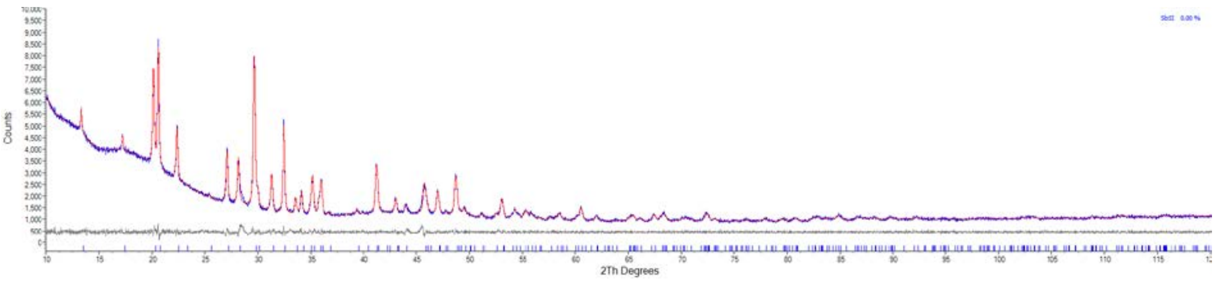

Figure S7. Fitted X-ray diffraction pattern of epoxy/SbSI nanocomposite under 400N load; blue line represent experimental data, red one – simulated curve, grey line – differential curve; blue ticks represents peaks position of orthorhombic SbSI Pnam structure

500N:

Phase name	SbSI
R-Bragg	1.967
Spacegroup	Pnam
Scale	0.0000722(3)
Cell Mass	2252(5)
Cell Volume (Å^3)	355.471(3)
Wt% - Rietveld	100.000
Double-Voigt|Approach	
Cry size Gaussian	88(2)
k: 1 LVol-IB (nm)	85(2)
k: 0.89 LVol-FWHM (nm)	83(2)
Strain	
Strain L	0.357(10)
e0	0.00076(2)
Crystal Linear Absorption Coeff. (1/cm)	2663(5)
Crystal Density (g/cm^3)	10.52(2)
Lattice parameters	
a (Å)	8.5392(4)
b (Å)	4.1019(2)
c (Å)	10.1492(5)

Site	Np	x	y	z	Atom	Occ	Beq
SB1	8	0.1234(3)	0.1073(9)	0.1212(3)	Sb	0.984(3)	0.5
S1	8	0.3446(11)	0.138(4)	0.4553(8)	S	0.984(13)	0.5
I1	8	0.0073(3)	0.0962(8)	0.6731(2)	I	1.025(3)	0.5

Rexp : 2.47 Rwp : 3.98 Rp : 2.81 GOF : 1.61

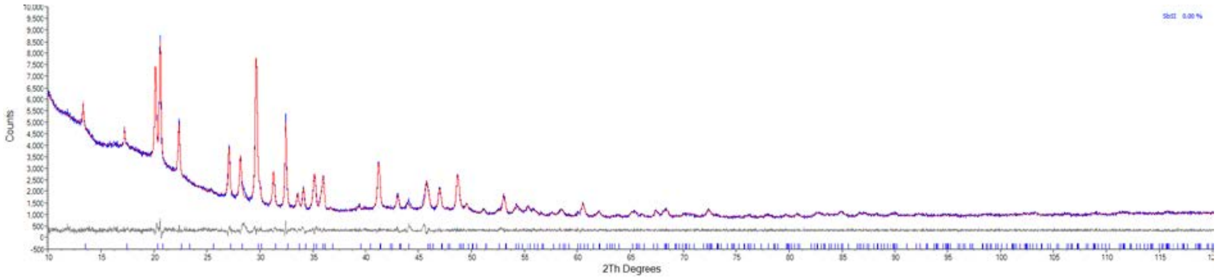

Figure S8. Fitted X-ray diffraction pattern of epoxy/SbSI nanocomposite under 500N load; blue line represent experimental data, red one – simulated curve, grey line – differential curve; blue ticks represents peaks position of orthorhombic SbSI Pnam structure

600N:

Phase name	SbSI
R-Bragg	2.057
Spacegroup	Pnam
Scale	0.0000748(3)
Cell Mass	2256(6)
Cell Volume (Å^3)	355.306(4)
Wt% - Rietveld	100.000
Double-Voigt|Approach	
Cry size Gaussian	84(3)
k: 1 LVol-IB (nm)	82(2)
k: 0.89 LVol-FWHM (nm)	79.9(2)
Strain	
Strain L	0.411(12)
e0	0.00094(3)
Crystal Linear Absorption Coeff. (1/cm)	2668(6)
Crystal Density (g/cm^3)	10.55(3)
Lattice parameters	
a (Å)	8.5391(5)
b (Å)	4.1018(3)
c (Å)	10.1426(6)

Site	Np	x	y	z	Atom	Occ	Beq
SB1	8	0.1232(3)	0.1124(10)	0.1221(3)	Sb	0.976(3)	0.5
S1	8	0.3446(11)	0.137(5)	0.4587(9)	S	1.007(14)	0.5
I1	8	0.0065(3)	0.0931(8)	0.6732(2)	I	1.032(3)	0.5

Rexp : 2.52 Rwp : 4.31 Rp : 3.05 GOF : 1.71

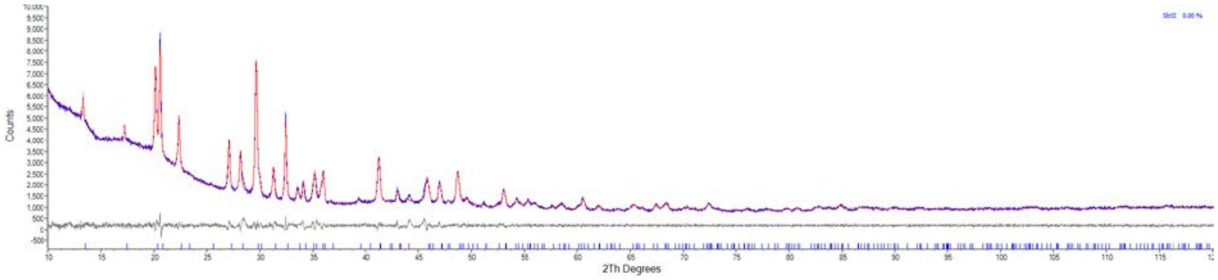

Figure S9. Fitted X-ray diffraction pattern of epoxy/SbSI nanocomposite under 600N load; blue line represent experimental data, red one – simulated curve, grey line – differential curve; blue ticks represents peaks position of orthorhombic SbSI Pnam structure

700N:

Phase name	SbSI
R-Bragg	1.999
Spacegroup	Pnam
Scale	0.0000716(5)
Cell Mass	2238(5)
Cell Volume (Å^3)	355.274(3)
Wt% - Rietveld	100.000
Double-Voigt|Approach	
Cry size Gaussian	86(2)
k: 1 LVol-IB (nm)	82(2)
k: 0.89 LVol-FWHM (nm)	78.5(2)
Strain	
Strain L	0.481(10)
e0	0.00074(2)
Crystal Linear Absorption Coeff. (1/cm)	2645.5(2)
Crystal Density (g/cm^3)	10.4317(9)
Lattice parameters	
a (Å)	8.5386(4)
b (Å)	4.1017(2)
c (Å)	10.1382(5)

Site	Np	x	y	z	Atom	Occ	Beq
SB1	8	0.1232(3)	0.1087(9)	0.1213(3)	Sb	0.984(3)	0.5
S1	8	0.3433(11)	0.136(4)	0.4516(8)	S	0.956(10)	0.5
I1	8	0.0077(3)	0.0948(8)	0.6731(2)	I	1.018(3)	0.5

Rexp : 2.44 Rwp : 3.85 Rp : 2.76 GOF : 1.58

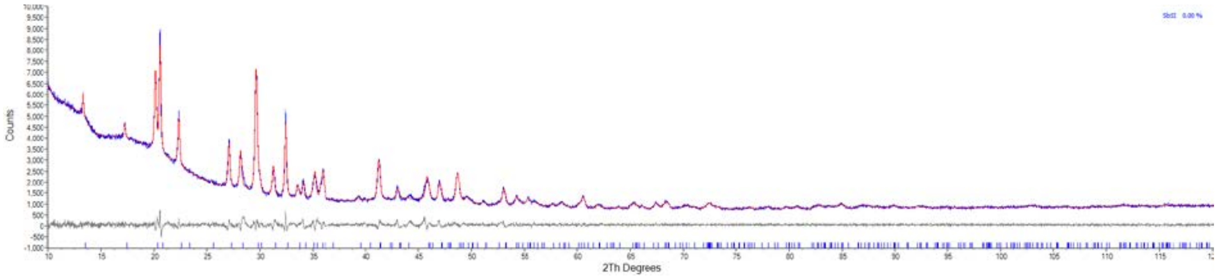

Figure S10. Fitted X-ray diffraction pattern of epoxy/SbSI nanocomposite under 700N load; blue line represent experimental data, red one – simulated curve, grey line – differential curve; blue ticks represents peaks position of orthorhombic SbSI Pnam structure
